# Identification and Characterization of *RcMADS1*, an *AGL24* Ortholog from the Holoparasitic Plant *Rafflesia cantleyi* Solms-Laubach (Rafflesiaceae)

**DOI:** 10.1371/journal.pone.0067243

**Published:** 2013-06-28

**Authors:** Rengasamy Ramamoorthy, Edwin Ek-Kian Phua, Saw-Hoon Lim, Hugh Tiang-Wah Tan, Prakash P. Kumar

**Affiliations:** 1 Department of Biological Sciences, Faculty of Science, National University of Singapore, Republic of Singapore; 2 Malaysia University of Science and Technology, Petaling Jaya, Selangor, Malaysia; 3 School of Biological Sciences, Monash University, Clayton, Victoria, Australia; 4 Temasek Life Sciences Laboratory, Research Link, National University of Singapore, Republic of Singapore; School of Biomedical Sciences, The University of Queensland, Australia

## Abstract

*Rafflesia*, a holoparasitic genus that produces the largest flower in the world is characterized by the absence of leaves, stem and other macroscopic organs. To better understand the molecular regulation of flower development in this genus we isolated and characterized a floral MADS-box gene, namely, *RcMADS1* from *Rafflesia cantleyi*. Heterologous expression analysis in *Arabidopsis* was chosen because *Rafflesia* is not amenable to genetic manipulations. *RcMADS1* shares sequence similarity with *AGAMOUS-LIKE 24* (*AGL24*) and *SHORT VEGETATIVE PHASE* (*SVP)* of *Arabidopsis*. Ectopic expression of *RcMADS1* in *Arabidopsis* caused early flowering and conversion of sepals and petals into leaf-like structures, and carpels into inflorescences. In *35S*::*RcMADS1* plants *SUPPRESSOR OF OVEREXPRESSION OF CONSTANS 1* (*SOC1*), a downstream target gene of *AGL24,* was upregulated. *35S*::*RcMADS1* plants exhibit early flowering and conversion of the floral meristem into inflorescence meristem, as in *35S*::*AGL24* plants. Similar to *AGL24*, *RcMADS1* could rescue the late flowering phenotypes of *agl24-1* and *FRIGIDA*, but not the early flowering of *svp-41*. Based on these results, we propose that *RcMADS1* is a functional ortholog of *Arabidopsis AGL24*.

## Introduction

**Figure 1:**
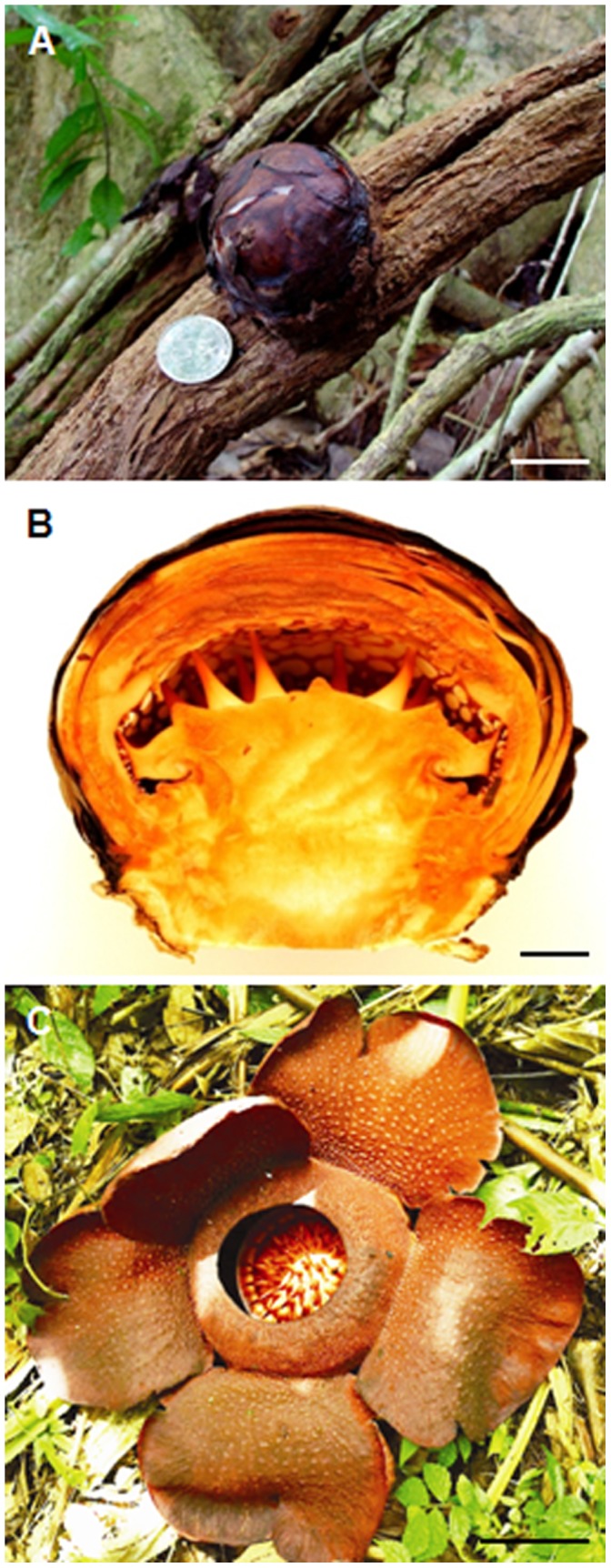
*Rafflesia cantleyi* Solms-Laubach buds and flower. (A) Bud of approximately 4 cm developing on a *Tetrastigma* sp. vine in the forest in Pulau Tioman, Malaysia. (B) A longitudinal section of an older bud approximately 8 cm in diameter. (C) An open flower of *Rafflesia* sp. (approximately 1 m in diameter). Scale bars A = 2 cm, B = 1 cm and C = 15 cm.

*Rafflesia* is a parasitic plant in a distinctive flowering plant genus from Rafflesiaceae that develops the largest flower in the world [1]. The members of this genus are unique among the flowering plants owing to their highly reduced vegetative morphology, prominent and large floral structures, and physiology. *Rafflesia* species are holoparasitic endophytes of *Tetrastigma* (Vitaceae). They lack visible leaves, stems and roots and only appear as flowers for sexual reproduction [2]. Their vegetative body is reduced to mycelium-like structure, which grows completely embedded within the host plants. Hence, they are fully dependent on their host for nutrition. Flowers of *Rafflesia cantleyi* are particularly distinctive compared to those of other species. The large fleshy flowers can reach up to one meter in diameter (Figure 1), and produce the smell of rotting flesh that attracts carrion flies for pollination [2]. *Rafflesia* flowers have some unusual structures, such as a modified perianth (perigone) enclosed by a diaphragm; a central column with an apical disk; and the presence of ramenta on the interior surface of the perigone tube and diaphragm (Figure S1) [3].

The habitat of *Rafflesia* is confined to the Indo-Malayan region [3]. In recent years, several new species have been discovered in the Philippines such as *Rafflesia speciosa* [4], *Rafflesia mira* [5], *Rafflesia baletei* [6], *Rafflesia lobata* [7] and *Rafflesia banahawensis* [8]. *Rafflesia* has not been well studied, with only a few reports published in the past (for example, [1], [9], [10]). The paucity of work on this genus is partly due to its rarity and inaccessibility of its habitats. Holoparasitic plants like *Rafflesia* have undergone many physiological and morphological adaptations during their evolution and have lost most of the vegetative plant structures. Based on the sequence of the mitochondrial gene *matR, Rafflesia* was phylogenetically placed under the order Malpighiales [1]. *Rafflesia* was later found to be nested in the Euphorbiaceae in a more restricted study of the Malpighiales using five mitochondrial genes (*ccmB, cob, matR, nad6* and *rps3*) and a chloroplast gene (*matK*) [11]. It is interesting to note that *Rafflesia* evolved from a family with very small flowers. It is worthwhile to study the molecular nature of floral development of *Rafflesia* because the flower is the only macroscopic structure of the plant, and the flowers are highly unusual (Figure 1). Such studies can shed light on the developmental and evolutionary processes that *Rafflesia* has undergone.

Many key processes in growth and development are regulated by transcription factors, which can be classified into gene families according to the conserved DNA-binding domain present. In plants, there are about 60 different transcription factor gene families [12], [13], [14]. The MADS-box gene family is one of the major transcription factor gene families and it is particularly important in controlling floral transition, floral meristem identity, floral organ specification, and fruit and ovule development [15], [16], [17].The MADS-box encodes a DNA-binding domain comprising of approximately 60 amino acids, and is highly conserved across plants, fungi, and animals [18]. MADS-box genes are classified as type I (comprising of the subfamilies Mα, Mβ and Mγ), and type II (consisting of subfamilies Mδ/MIKC* and MIKC^C^) [19], [20], [21]. Almost all plant MADS-box genes that have been characterized so far belong to MIKC^C^ subfamily, with a modular structure comprising of four domains, namely, the MADS (M), intervening (I), keratin-like (K), and C-terminal (C) domains [18], [19], [22]. Additionally, based on their functions and expression patterns the MADS-box genes involved in regulating floral development are grouped under classes A, B, C, D, E, F, G, Bs (B-sister) and T [23].

Flowering is controlled by an intricate network of signaling pathways, which are regulated by environmental and developmental cues [24]. Five different pathways, namely, vernalization, photoperiod, gibberellin, autonomous and aging pathways are involved in this process [25], [26]. Many genes characterized in these pathways were MADS-box genes, which include floral organ-identity (eg. *AGAMOUS* and *SEPALLATA 1, 2, 3* and *4*) and flowering-time genes, such as *AGL24* [27], [28], [29], [30] and *SVP* [31], [32]. AGL24 and SVP are members of the StMADS11 clade [22], [31], and are involved in the contrasting functions of promotion and repression of flowering, respectively. AGL24 promotes flowering by inducing expression of *SOC1* by directly binding to its promoter [30]. In contrast, SVP suppresses the expression of *SOC1* by directly binding to its promoter [33]. These observations clearly show that AGL24 and SVP are key integrators of flowering signals, along with other floral transition signals [30].

Overexpression of *AGL24* in *Arabidopsis* results in early flowering and floral abnormalities such as, secondary flowers in the axils of leaf-like sepals of primary flowers, elongation of the base of ovaries into inflorescence stem-like structures, and production of ectopic inflorescences from swollen ovaries [27], [32]. In contrast, overexpression of *SVP* results in late flowering and loss of carpels as well as the conversion of flowers into shoot-like structures with chimaeric characteristics of vegetative shoots and flowers [32]. Homologues of *AGL24* and *SVP* have been isolated from *Antirrhinum* [34], *Brassica* [35], barley [36], rice [37], [38], [39], tomato [40] and *Withania* [41]. When they are ectopically expressed in *Arabidopsis*, phenotypes are similar to either *35S::AGL24* [28] or *35S::SVP* [32] plants with altered flowering time and floral organ defects. This shows that AGL24 and SVP are likely to have a conserved function in specifying floral meristem development among most of the flowering plants.

Here we report the isolation, molecular and functional characterization of a MADS-box gene named as *RcMADS1* from *Rafflesia cantleyi*. The full-length cDNA was cloned using a reverse transcription-PCR approach. This cDNA shows high sequence similarity to several MADS-box genes, in particular, to *AGL24* and *SVP* of *Arabidopsis thaliana.* Ectopic expression of this gene (*35S::RcMADS1*) in *Arabidopsis* resulted in altered flowering time and a flower morphology phenotype similar to that of *35S::AGL24*. Expression analysis of *SOC1,* one of the downstream target genes of *AGL24* and *SVP*, was performed by quantitative Real-Time Polymerase Chain Reaction (qRT-PCR) in transgenic lines harboring *35S::RcMADS1*. Functional complementation and rescue analysis were performed using late flowering *agl24-1*, early flowering *svp-41* and late flowering *FRIGIDA (FRI)*. Our data regarding flowering time, floral phenotype, complementation, rescue and *SOC1* expression analysis suggest that *RcMADS1* is a functional ortholog of *AGL24*. Also, despite the highly specialized evolutionary reduction of vegetative parts in *Rafflesia* the molecular regulation of flowering may be conserved between *Arabidopsis* and *Rafflesia*.

## Materials and Methods

### Plant Materials

Flower buds of various sizes of *Rafflesia cantleyi* Solms-Laubach were collected from several localities in Pulau Tioman, Pahang, Malaysia (Permission from the Economic Planning Unit, Prime Minister’s Office, Malaysia - project reference no. UPE:/40/200/19 SJ. 1200, and research pass no.1163). The buds were surface-sterilized using a 10% (v/v) Clorox® (1% sodium hypochlorite) for 5–10 min, followed by rinsing with sterile water. Tissues were cut and weighed, then flash-frozen in liquid nitrogen. All samples were stored at –80°C.

Transgenic and mutant *Arabidopsis thaliana* plants used in the experiments were of the same genetic background, i.e., Columbia ecotype. *Arabidopsis thaliana* seeds were sown on soil (Flora Fleur) and stratified for 3–4 days at 4°C, before being transferred to a growth chamber maintained at 22±2°C under 16 h of light/8 h of dark photoperiod.

### RNA Extraction

Total RNA from the *Rafflesia cantleyi* flower buds was isolated using a modified RNeasy® Plant Mini Kit (QIAGEN) method [42]. The modification involves an initial CTAB extraction [43]. The extract was then applied to an RNeasy® column and purified following the manufacturer’s instructions.

Total RNA from *Arabidopsis thaliana* plant tissues was isolated using the RNeasy® Plant Mini Kit (QIAGEN) following the manufacturer’s instructions.

### Reverse Transcription

First strand cDNA synthesis was carried out from 2 µg of total RNA using Maxima® First Strand cDNA Synthesis Kit (Fermentas, Cat.No.K1641) as per the manufacturer’s protocol.

### PCR Amplification

PCR amplification of MADS-box genes from *Rafflesia cantleyi* cDNA was performed using degenerate primers and an oligo (dT)_15_ primer. These degenerate primers were designed based on the conserved MADS box of MADS box genes. The primers used were forward: 5′-GGGGTACCAAYMGICARGTIACITAYTCIA AGMGIMG-3′, reverse: PY1: GAGAGAGAGAGAACTAGTCTCGAGTTTTTTT TTTTTTTT. PCR reactions were performed using step-up conditions with the following cycling parameters: an initial denaturation at 95°C for 1 min; 10 cycles of denaturation at 95°C for 30 s, annealing at 35°C for 1 min, and extension at 72°C for 1 min; 25 cycles of denaturation at 95°C for 30 s, annealing at 40°C for 1 min, and extension at 72°C for 1 min; and a final extension at 72°C for 10 min. 1 µg of cDNA template was added to a reaction mixture consisting of 1× DyNAzyme PCR buffer, 0.2 mM dNTP mix, and 2 pmole each of forward and reverse primers and DyNAzyme polymerase. PCR reactions were visualized by performing gel electrophoresis in a 1.2% agarose gel. Amplified fragments over 400 bp in size were selected for cloning and sequencing.

### Cloning, DNA Sequencing and Sequence Analysis of PCR Products

The PCR products were purified using the QIAquick® PCR purification kit (QIAGEN) following the manufacturer’s instructions. The purified PCR product was cloned into the pGEM®-T Easy Vector (Promega). Selected clones were sequenced via an automated sequencing method using ABI PRISM™ Big Dye™ Terminator Cycle Sequencing Ready Reaction Kit (Applied Biosystems). Sequences obtained after automated sequencing were collated and compared with published sequences in the GenBank database using the Basic Local Alignment Search Tool (BLAST) program on the National Center for Biotechnology Information (NCBI) website. The algorithms used were blastn (to search the nucleotide database using a nucleotide query) and tblastx (to search the translated nucleotide database using a translated nucleotide query).

### RACE

Complete cDNAs were obtained using BD SMART™ RACE cDNA Amplification Kit (Clontech) following the manufacturer’s instructions. The full length cDNA sequence of *RcMADS1* was submitted to GenBank database and the accession number is KC894756.

### Phylogenetic Analysis

Phylogenetic analysis was performed using the Phylogeny.fr online software (http://www.phylogeny.fr/version2_cgi/simple_phylogeny.cgi) with one click mode, where MUSCLE, Gblocks, PhyML, and TreeDyn programs were used for multiple alignment, curation, tree building and tree rendering, respectively [44].

### Construction of *RcMADS1* Ectopic Expression Transgenic *Arabidopsis thaliana* Lines

The full open reading frame of the *RcMADS1* cDNA from *Rafflesia cantleyi* was amplified using the following primers containing restriction enzyme sites RcMADS1-F-BamHI 5′-GGATCCATGGCTCGAGAAAAGATCAA-3′, and RcMADS1-R-SpeI 5′-ACTAGTGCTTGAGAAGGACAATCCC-3′. The PCR products were purified using the QIAquick® PCR purification kit (QIAGEN) following the manufacturer’s instructions. The purified PCR product was cloned into pGEM®-T Easy Vector (Promega). Resultant plasmids were digested for 2 h with BamHI and SpeI restriction enzymes to release the *RcMADS1* fragment, which was then inserted in between the CaMV *35S* promoter and terminator in a sense direction into the pGreen0229 vector [45]. This ectopic expression construct was named *35S::RcMADS1.* To generate *AGL24::RcMADS1* construct ∼3.2 kb genomic fragment of *AGL24* promoter region was cloned, which includes the 1^st^ exon and intron (it was reported that 1^st^ intron of *AGL24* has two CArG-box *cis*-elements, which is important for its regulation by upstream transcription factors) [32], Transformation of *Arabidopsis thaliana* plants was carried out using the floral dipping method as described by Clough and Bent, 1998 [46]. Healthy *Arabidopsis thaliana* plants of WT, *agl24-1*, *FRI-*containing Col line and *svp-41* were grown on soil under long-day photoperiod conditions (16 h of light/8 h of darkness), until flowering. The seeds were harvested following floral dip transformation and screened for herbicide resistance. The seedlings were grown under long-day conditions and sprayed with 250 mg/l Basta® solution (Finale, AgrEvo, California, USA) 5 days and 10 days after germination. After 2 weeks, seedlings were examined for resistance against the herbicide. The *agl24-1* and *svp-41* mutant seeds were kindly provided by Dr. Hao Yu, Department of Biological Sciences, National University of Singapore (DBS, NUS) and *FRI-*containing Col line seeds were kindly provided by Dr. Yuehui He, DBS, NUS.

### Quantitative Real-Time PCR

Quantitative Real-Time Polymerase Chain Reaction (qRT-PCR) analyses were performed using Applied Biosystems (ABI) StepOne™ Real-Time PCR System with denaturation at 95°C for 10 min, followed by 40 cycles of denaturation at 95°C for 15 s and annealing/extension at 60°C for 1 min. Triplicate quantitative assays were performed on 1 µl (∼20 ng) of each cDNA dilution using the Fast SYBR® Green Master Mix (ABI, P/N 4385612). Primers used for this are *SOC1-*F*-*5′-AGC TGCAGAAAACGAGAAGCTCTCTG-3′, *SOC1-*R-5′GGGCTACTCTCTTCATC ACCTCTTCC-3′, *TUB2* gene used as endogenous control *TUB2*-F-5′AAGGACCT ACTTCGGTGATGAG-3′,*TUB2*-R*-*5′GCTCTCCACCAATGTTAAGATGAG-3′. Two biological replicates each with three technical replicates were used and relative expression levels were calculated as previously described [29].

## Results

### Isolation and Sequence Analysis of a MADS-box Gene from *Rafflesia cantleyi*

We succeeded in cloning a MADS-box gene by reverse-transcription PCR using degenerate primers with cDNA synthesized from *Rafflesia cantleyi* floral buds. One fragment of 750 bp from the initial round of PCR contained a MADS-box gene that was named *RcMADS1* (for *Rafflesia cantleyi* MADS-box1). Using 5′ Rapid Amplification of cDNA Ends (5′ RACE) followed by amplification with gene-specific primer and oligo-dT primer, we obtained an 899 bp long sequence. This cDNA sequence contains a 687 bp long open reading frame (ORF) including a stop codon, which encodes a polypeptide of 228 amino acids, as well as 5′ and 3′ untranslated regions (Figure S2). Results from a tblastx search using the online BLAST program revealed high sequence similarities to MADS-box proteins such as PtMADS1 from *Populus tomentosa*, JOINTLESS from *Solanum lycopersicum*, MPF2 from *Physalis pubescens*, MPP3 from *Physalis peruviana*, IbMADS4 from *Ipomoea batatas*, StMADS11 and StMADS16 from *Solanum tuberosum*, and SVP and AGL24 from *Arabidopsis thaliana*, all from the StMADS11 clade of MADS-box genes. An alignment of these proteins with RcMADS1 showed that the MADS-box and ‘K’ domain are highly conserved amongst these members of the StMADS11 clade, with the ‘I’ domain somewhat well conserved as well. The MADS domain (69 amino acids) was identified using the NCBI conserved domain search, while comparison with known MADS-box genes allowed the identification of the K-box consisting of 79 amino acid residues (Figure S3).

### Phylogenetic Analysis of RcMADS1

**Figure 2:**
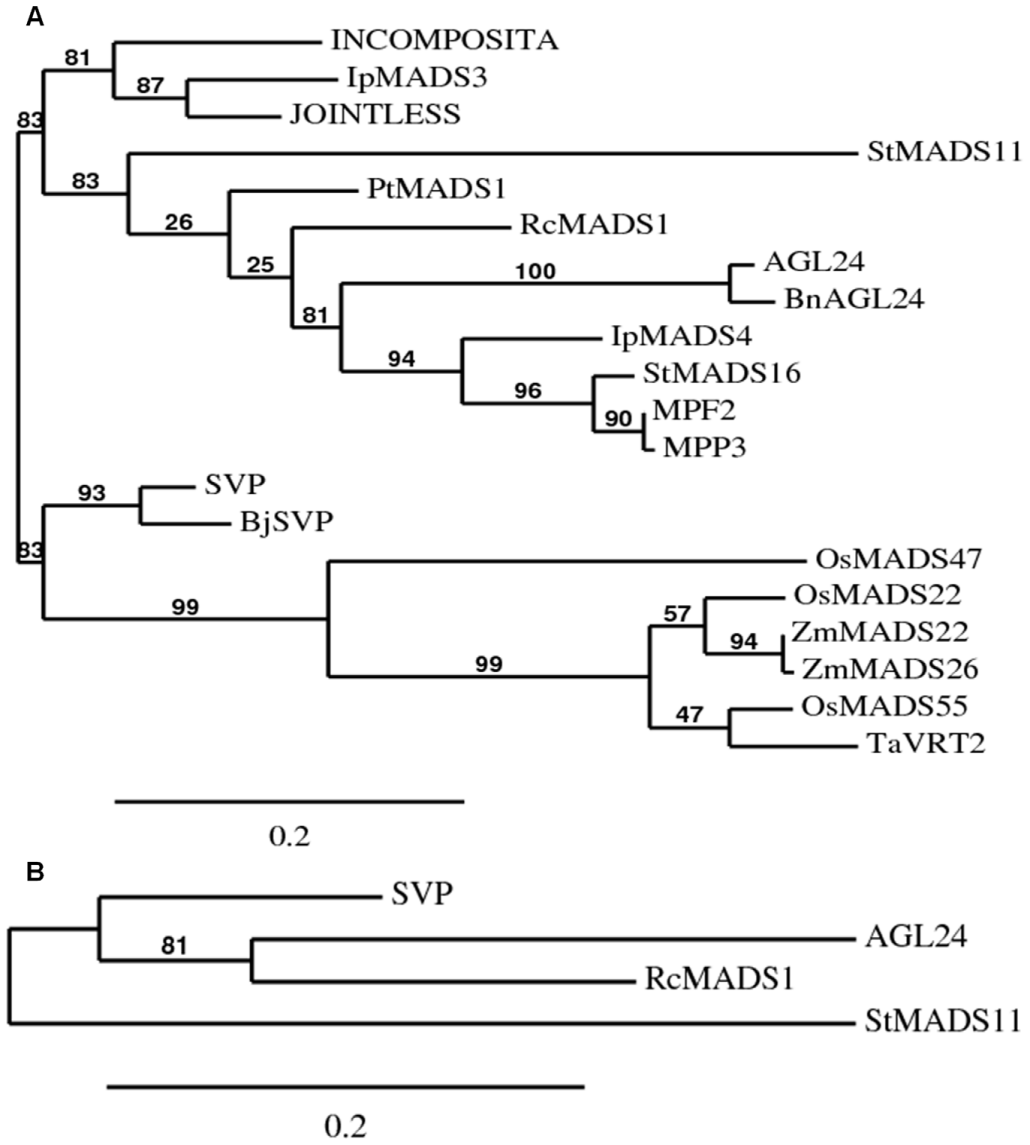
Phylogenetic tree of selected MADS-box genes from StMADS11 clade. (A) The tree was constructed based on the deduced amino acid sequences using the Phylogeny.fr with one click mode. (B) Phylogenetic tree of RcMADS1 with only AGL24, SVP and StMADS11. In both the trees RcMADS1 is nested closer to AGL24 than to SVP. AGL24 and SVP from *Arabidopsis thaliana*; BnAGL24 from *Brassica napus*; BjSVP from *Brassica juncea*; INCOMPOSITA from *Antirrhinum majus*; IbMADS3 and IbMADS4 from *Ipomoea batatas*; JOINTLESS from *Solanum lycopersicum*; MPF2 from *Physalis pubescens*; MPP3 from *Physalis peruviana*; OsMADS22, OsMADS47, OsMADS55 from *Oryza sativa*; PtMADS1 from *Populus tomentosa*; RcMADS1 from *Rafflesia cantleyi*; StMADS16 and StMADS11 from *Solanum tuberosum*; TaVRT2 from *Triticum aestivum*; ZmMADS22 and ZmMADS26 from *Zea mays*. The numbers next to the nodes are bootstrap percentages. The scale bars denote a divergence of 0.2 amino acid substitutions per site.

Phylogenetic analysis of amino acid sequences derived from members of the StMADS11 clade showed that RcMADS1 is nested within the StMADS11 clade, and fell into a clade with AGL24, to the exclusion of SVP, with 83% bootstrap support (Figure 2A). When we used RcMADS1 with only StMADS11, AGL24 and SVP amino acid sequences, it still forms a clade with AGL24 distinct from SVP (Figure 2B). Phylogenetic analysis of derived amino acid sequences from the conserved MADS-box and K-box also showed that RcMADS1 groups with AGL24 (Figure S4A and B). When a more extensive phylogenetic tree was constructed using the conserved MADS-box domain of the StMADS11 clade and representative members of other MADS-box gene clades, RcMADS1 remained in the StMADS11 clade, which shows that despite high sequence similarities, RcMADS1 is closer to AGL24 than to SVP (Figure S5).

### Ectopic Expression of *RcMADS1* in *Arabidopsis* Displayed Abnormal Floral Organs and Early Flowering

In order to understand whether RcMADS1 is functionally related to AGL24 or SVP, we generated transgenic *Arabidopsis thaliana* lines. Since *Rafflesia* is a holoparasite that lacks vegetative parts, the use of the heterologous system is the best approach for functional analyses. A total of 23 independent transgenic *Arabidopsis* lines harboring *35S::RcMADS1* were generated using the floral dip method [46].

**Figure 3:**
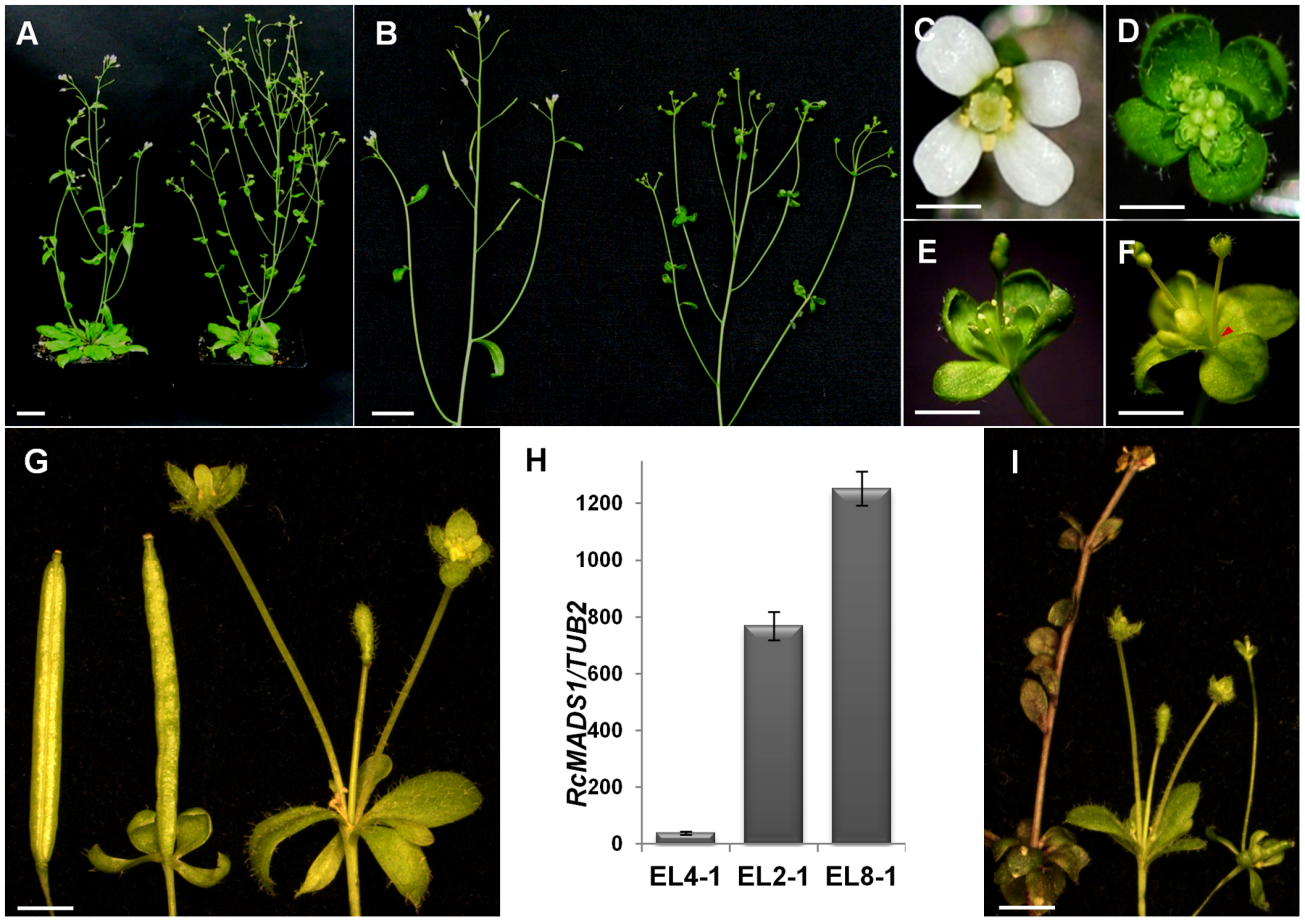
Ectopic expression of *RcMADS1* in *Arabidopsis thaliana* causes conversion of carpels into inflorescence-like structures. The stronger phenotypic lines are shown (A) Comparison of *35S::RcMADS1* (right) with WT (left). (B) Close-up of one inflorescence stem of *35S::RcMADS1* (right) and WT (left). (C). A wild-type *Arabidopsis* flower. (D) A *35S::RcMADS1* flower showing conversion of sepals and petals into leaf-like structures bearing conspicuous trichomes. (E) A *35S::RcMADS1* flower showing the conversion of carpel into an inflorescence-like structure. (F) A *35S::RcMADS1* flower with a secondary inflorescence developing from the axil of a sepal (red arrowhead). (G) *35S::RcMADS1* flowers displaying the three different severities of phenotypes. Weak: flower from EL4 similar to wild-type (left), Medium: flower from EL2 exhibiting conversion of sepals and petals into leaf-like structures while the carpel develops into a silique (middle), Strong: flower from EL8 exhibiting conversion of sepals and petals into leaf-like structures and the carpel is converted into inflorescence-like structure with secondary inflorescences (right). (H) Expression levels of *RcMADS1* in the three different transgenic lines exhibiting weak, medium and strong phenotypes. Expression levels were normalized against the expression of *TUB2*. Error bars indicate s.d. (I) Flowers of *35S::AGL24* (right), *35S::RcMADS1* (middle) and *35S::SVP* (left). Scale bars A, B = 1cm, C, D, E, F = 1 mm, G and I = 2 mm.

The *35S::RcMADS1* plants showed three distinct phenotypes. Five out of the 23 transgenic lines had a ‘strong’ phenotype where plants produced more inflorescence axes compared to wild-type plants (Figure 3A and B) and exhibited sepals and petals into leaf-like structures bearing conspicuous trichomes (Figure 3C and D). Additionally, instead of carpels at the center, elongated inflorescence-like structures developed (Figure 3E and F). The late-formed flowers produced fertile siliques, but containing only a few viable seeds (Figure S6A, B and C). Four lines had a ‘weak’ phenotype where only leaf-like structures were formed with trichomes where the sepals should be, but the carpels developed as siliques and set viable seeds in the weak phenotype plants (Figure S7). The remaining transgenic lines exhibited phenotypes similar to that of wild-type plants. *RcMADS1* expression analysis in one line from each phenotype pool by qRT-PCR showed a direct correlation between the level of *RcMADS1* expression and phenotypic severities. Expression level was high in plants exhibiting the strong phenotype, and proportionately lower in lines with weak or no obvious changes in phenotype (Figure 3G and H). Even though *RcMADS1* sequence had high similarities to both *AGL24* and *SVP*, the ‘strong’ phenotype exhibited by plants ectopically expressing *RcMADS1* -was similar to that of *AGL24* ectopic expression phenotype [27], [28] and distinct from *SVP* ectopic expression [32] in *Arabidopsis thaliana* (Figure 3I).

**Figure 4:**
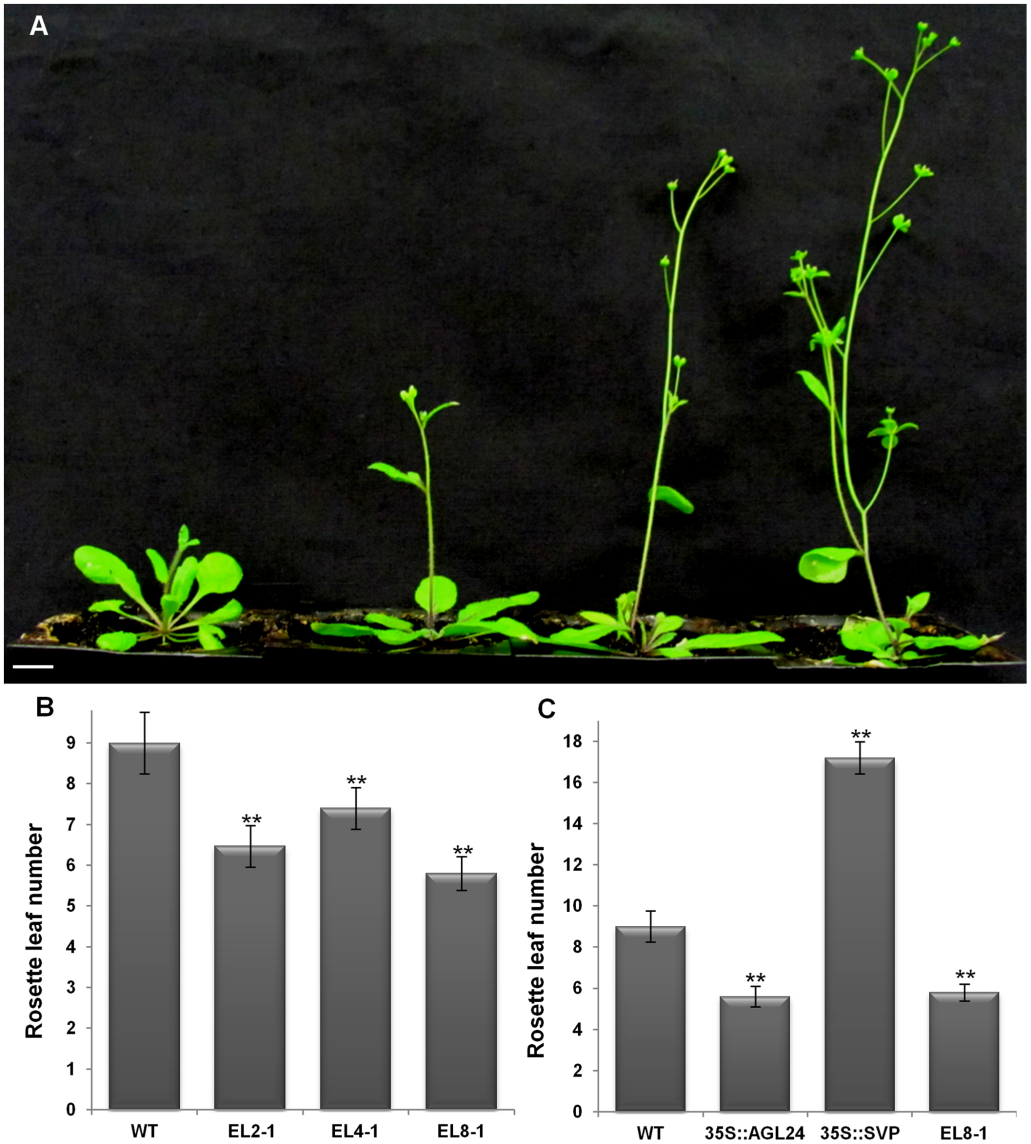
Ectopic expression of *RcMADS1* causes early flowering in *Arabidopsis*. (A) Representative plants from independent transgenic lines showing different flowering times (plant1 WT, plant2 EL4, plant3 EL2 and plant4 EL8). (B) The flowering time of plants in (A) indicated by rosette leaf numbers at bolting. Data are mean ± s.d. from 20 plants of each line. (C) Flowering time comparison of EL8 with WT, *35S::AGL24* and *35S::SVP.* Data are mean ± s.d. from 20 plants of each genotype. Asterisks indicate significantly different means (P≤0.05) according to Student’s *t*-Test. Scale bar = 1cm.

Ectopic expression lines (ELs) also showed an altered flowering time compared to wild-type (WT) plants as determined by rosette leaf numbers at the time of bolting. The ELs displayed an early flowering phenotype and a gradual decrease in the number of rosette leaves according to the phenotype severities. Three of the representative transgenic lines showing weak phenotype (EL2), no change (EL4) or with strong phenotype (EL8) were chosen for further analyses. Even though EL4 plants were similar to the WT they flowered earlier than the WT. The EL2 line flowered earlier than EL4, and EL8 flowered earlier than EL2 plants. The flowering time of ELs were also correlated to the *RcMADS1* expression levels and the phenotypic severities (Figure 4A and B). Thus, the flowering time of EL8 plants resembled the early flowering *35S::AGL24* plants rather than the late flowering *35S::SVP* plants (Figure 4C). The ectopic expression floral phenotypes and flowering time strongly suggested that RcMADS1 is functionally similar to AGL24.

### *RcMADS1* can Functionally Complement the *agl24-1* Mutant and Rescue the *FRI* Late Flowering Phenotypes

**Figure 5:**
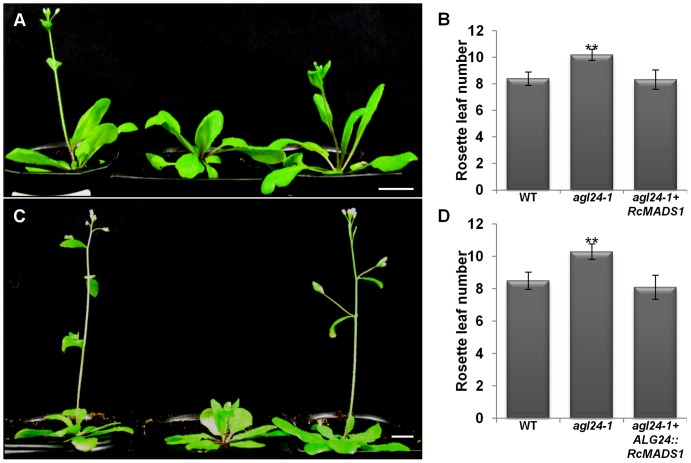
Complementation analysis of *agl24-1* mutant by *RcMADS1* expression driven by two different promoters. (A) Functional complementation of *agl24-1* by *35S::RcMADS1*. WT (left), *agl24-1* mutant (middle) and *agl24-1+35S::RcMADS1* (right). (B) The flowering time of plants in (A) represented by rosette leaf numbers at bolting (mean ± s.d.). (C) Functional complementation of *agl24-1* by *AGL24::RcMADS1*. WT (left), *agl24-1* mutant (middle) and *agl24-1+ AGL24::RcMADS1* (right). (D) The flowering time of plants in (C) represented by rosette leaf numbers at bolting (mean ± s.d.). Asterisks indicate that means are significantly different (P≤0.05) according to Student’s *t*-Test. Scale bars = 1cm.

**Figure 6:**
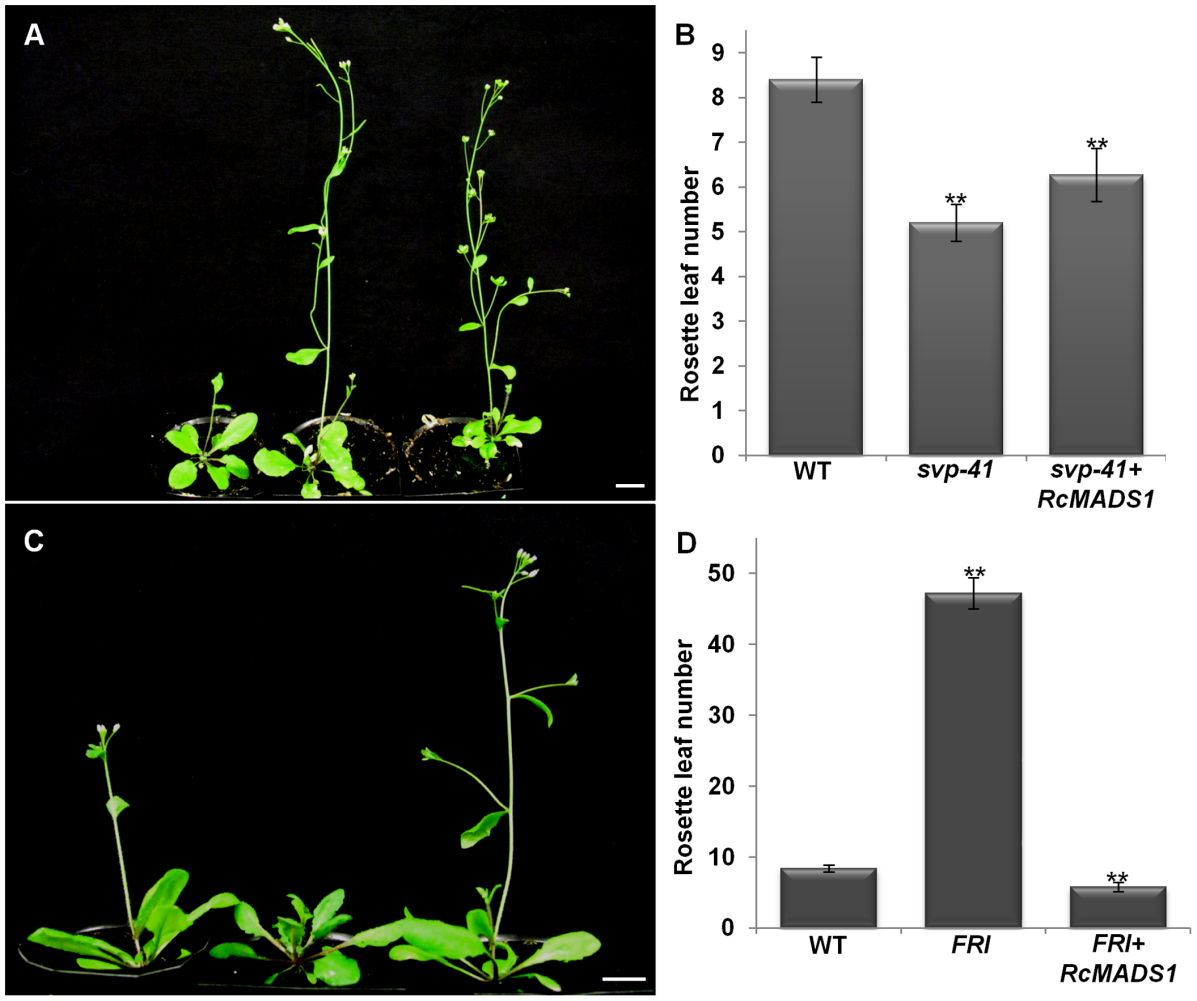
Complementation analysis of ***svp-41*** **mutant and rescue of** ***FRI*** **phenotype by** ***RcMADS1*** **expression.** (A) *RcMADS1* failed to rescue *svp-41*. WT (left), *svp-41* mutant (middle) and *svp-41+RcMADS1* (right). (B) The flowering time of plants in (A) represented by rosette leaf numbers at bolting (mean ± s.d.). (C) Rescuing of *FRI* phenotype. WT (left), *FRI* containing Col line (middle) and *FRI* containing Col line *+RcMADS1* (right). (D) The flowering time of plants in (C) represented by rosette leaf numbers (mean ± s.d.). Asterisks indicate that means are significantly different (P≤0.05) according to Student’s *t*-Test. Scale bars = 1cm.

To further verify RcMADS1 function, we made use of the late flowering *agl24-1, FRI-*containing Col line and the early flowering *svp-41* mutants. We generated transgenic plants expressing *RcMADS1* under the *35S* promoter or *AGL24* promoter in these three genotypes by floral dipping. At least three independent lines showing the phenotype were considered for all the complementation analyses. Plants ectopically expressing *RcMADS1* in the *agl24-*1 background flowered earlier than the *agl24-1* mutant (Figure 5A and B). When *RcMADS1* is driven by *AGL24* promoter, regulation of *RcMADS1* expression should be similar to that of native *AGL24*. Transgenic *agl24-1* plants expressing *AGL24::RcMADS1* also flowered earlier than the *agl24-1* mutant (Figure 5C and D), indicating that *RcMADS1* is able to rescue the late flowering phenotype of *agl24-1* with both the promoters used. On the other hand, the *svp-41* early flowering phenotype was not affected by *RcMADS1* ectopic expression and transgenic plants exhibited floral abnormalities such as conversion of sepals and petals into leaf-like structures (Figure 6A and B). In *Arabidopsis*, natural allelic variation at the *FRI* locus is one of the major determinants for flowering time and the dominant alleles of *FRI* confer late flowering, which can be reversed to early flowering by vernalization [25], [47]. The late flowering phenotype of *FRI* locus also can be rescued by *AGL24* overexpression in *Arabidopsis* [28]. Hence, we wanted to study the functional similarities of *RcMADS1* to *AGL24* by generating transgenic plants ectopically expressing *RcMADS1* in the late flowering *FRI-*containing Col line. These transgenic plants displayed an early flowering phenotype when compared with the *FRI*-containing Col line and WT (Figure 6A and B). Therefore, RcMADS1 could rescue the late flowering phenotype of the *FRI*-containing Col line, similar to AGL24. These observations further indicate that RcMADS1 functions like AGL24 in *Arabidopsis* plants.

### *RcMADS1* can Induce the Expression of *SOC1*, a Direct Downstream Target of AGL24

**Figure 7:**
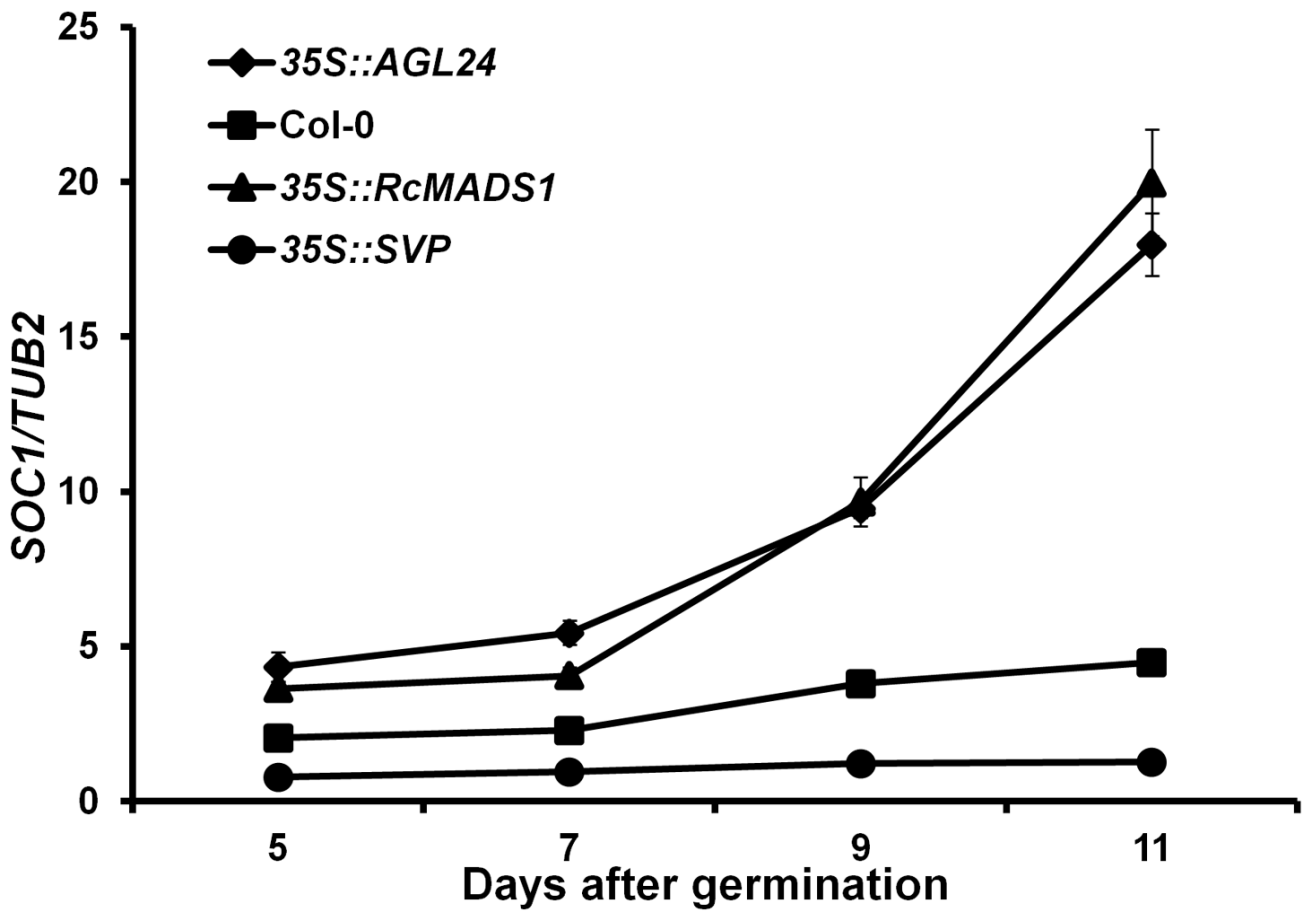
*SOC1* expression is up regulated by RcMADS1 during floral transition. The expression levels of *SOC1* in different genotypes (mean ± s.d.) during floral transition. Results were normalized against the expression of *TUB2*.

Functional complementation analyses in a heterologous system still pose the question of whether the molecular regulation of flowering time by the transgene is similar to that of AGL24. *SOC1* is known to be one of the direct downstream targets of both AGL24 and SVP, but AGL24 promotes *SOC1* expression while SVP represses it. Furthermore, according to earlier reports changes in *SOC1* expression can be detected during floral transition. Therefore, in order to gain insight into the molecular function of RcMADS1, we tested the expression of *SOC1* in the *35S::RcMADS1* transgenic line showing the strong phenotype along with WT, *35S::AGL24*, and *35S::SVP* seedlings as control. Expression of *SOC1* was analyzed by qRT-PCR using RNA from various seedling stages (5 to 11 days after germination, on alternate days). Our qRT-PCR results revealed that *SOC1* expression was significantly elevated in *35S::AGL24* and *35S::RcMADS1* plants compared to those in *35S::SVP* and WT seedlings 9 days after germination (Figure 7), which corresponds to the floral transition stage of development. This observation further showed that the molecular function may be conserved between RcMADS1 and AGL24 with respect to regulation of floral transition.

## Discussion

*Rafflesia* species are well known for their conspicuous flowers and holoparasitic endophytic nature that lack vegetative body parts such as leaves, roots, and stems [2]. Hence, flowers are the only plant parts amenable for analyses in this plant species. In this study we chose to study MADS-box genes, which are known to be involved in regulating floral organ identity and floral transition [15], [16], [17], [22], [28]. Using degenerate primers designed based on the conserved MADS-box region sequences, we could clone a full length MADS-box gene from *Rafflesia cantleyi* and named it *RcMADS1.* It showed high level of sequence similarity to the *Arabidopsis* MADS-box genes *AGL24* and *SVP*. Phylogenetic analysis of RcMADS1 showed that it might be closer to AGL24 than to SVP (Figure 2, Figure S4 and S5). In *Arabidopsis*, AGL24 is one of the integrators of flowering signals leading to a precise regulation of floral meristem specification [48], which occurs in a dosage-dependant manner [27]. *RcMADS1*, a putative ortholog of *AGL24* from *Rafflesia* may have a similar function. Similarly, ectopic expression of StMADS11 clade genes from other species in *Arabidopsis* caused alteration in flowering time and floral morphology, e.g., *BM1* from *Hordeum vulgare* [36] and *INCO* from *Antirrhinum majus* [34] in *Arabidopsis* caused the conversion of sepals and petals to leaf-like organs and the production of secondary inflorescence from axils of carpel. Likewise *OsMADS22, OsMADS47* and *OsMADS55* from *Oryza sativa* ectopically expressed in *Arabidopsis* showed floral abnormalities [37], [39] and *WAS206* (*MPF2*-*like* MADS-box gene related to *AGL24*) from *Withania* ectopic expression displayed early flowering in *Arabidopsis* [41].

Owing to the highly reduced nature of *Rafflesia*, we chose to use the heterologous model plant *Arabidopsis thaliana* for our studies. Transgenic *Arabidopsis* plants expressing *RcMADS1* allowed us to perform functional analyses that would be otherwise impossible to conduct with holoparasitic species. Phenotypic characterization of five independent transgenic lines expressing *RcMADS1* showed that plants were early flowering and exhibited conversion of the floral meristem into inflorescence meristems as seen in *35S::AGL24* plants [27], [28]. This is distinct from *35S::SVP* lines, which are characterized by late flowering and conversion of the inflorescences into chimeric shoot-like structures [48]. Moreover, the effect of *RcMADS1* ectopic expression is dosage-dependant, as is the case for *AGL24* [27], which was confirmed by the observed range of phenotypic severity seen in the various lines with differing levels of *RcMADS1* expression. These observations collectively suggest that *RcMADS1* might be a functional ortholog of *AGL24* in *Rafflesia cantleyi*. To further verify this notion, we performed complementation analysis using *agl24-1* and *svp-41* mutants. Our results showed that *RcMADS1* expression could functionally complement the loss of AGL24, but not SVP. Furthermore, we showed that *RcMADS1* is able to rescue the late flowering phenotype of *FRI* containing Col line, similar to the activity of *AGL24* [28]. Finally, regulation of expression of a direct downstream target of AGL24, namely *SOC1* by *RcMADS1* further supports the argument that *RcMADS1* is an ortholog of *AGL24* from *Rafflesia cantleyi.* Expression levels of *SOC1* were previously shown to be antagonistically regulated by AGL24 and SVP [33], [48] and our results showed that *RcMADS1* has the same effect on *SOC1* expression as *AGL24*. Taken together, our data show that RcMADS1 is a functional ortholog of *Arabidopsis* AGL24. The expression of *SOC1* in seedlings undergoing floral transition implies that signals necessary for activation of the upstream regulator, namely, AGL24, and the functional ortholog RcMADS1 in this case, are perceived and processed by the vegetative organs of the plant. Despite the fact that in *Rafflesia* these vegetative organs are absent, the function of RcMADS1 appears to be conserved. It is tempting to speculate that the flowering signals that usually originate from the vegetative parts of the plant e.g., florigen [49], [50], [51] may be provided by the host plant in holoparasitic species such as *Rafflesia*. Also, it should be noted that structurally similar proteins may behave differently in heterologous systems. Likewise RcMADS1 may function differently in *Rafflesia* than in *Arabidopsis*, which is yet to be studied. Although the rest of the mechanism of regulation of the process is not understood at this time, our study suggests that the molecular regulation of flowering in this species may be well conserved.

## Supporting Information

Figure S1 **Different floral parts of** ***Rafflesia*** ** ***micropylora*** **Meijer.** (a) Flower with the small aperture in the diaphragm. (b) Median longitudinal section view of female flower showing central column (ap: aperture in the diaphragm; coll: collum, neck of column; cup: cupula, perigone tube; ov: ovary; proc: processi on apex of disc; ram: ramenta on inside of cupula and diaphragm; sul: sulcus under disc. (c) Side view of the column showing outer and inner annulus (ae: annulus exterior; ai: annulus interior). (d) Section of male flowers, anther in longitudinal section and seen from lower side of the overhang of the ‘corona’ of the disc towards the sulcus. (e) Details of ramenta, often branched with swollen apices. This Figure is used with permission from Flora Malesiana Ser. I. Vol. 13 (1997).
(TIF)

Figure S2 **Sequence of** ***RcMADS1*** **cDNA.** The upper row is the nucleotide sequence, and the deduced amino acid sequence is in the lower row. The translation start (ATG) and termination (TGA) codons are underlined. The MADS-box and K domains are shown in red and green color, respectively.
(TIF)

Figure S3 **Alignment of the derived amino acid sequences of** ***RcMADS1*** **and other members of the** ***StMADS11*** **clade.** The MADS, I, and K domains are all relatively conserved across the various proteins. Identical residues are coloured dark blue. Key to sequences included: RcM1 = *RcMADS1* from *Rafflesia cantleyi*; MPF2 from *Physalis pubescens*; MPP3 from *Physalis peruviana*; *AGL24* and *SVP* from *Arabidopsis thaliana*; and *StMADS16* and *StMADS11* from *Solanum tuberosum*.
(TIF)

Figure S4 **Phylogenetic tree of selected MADS-box genes from StMADS11 clade.** (A) Phylogenetic tree showing clustering of RcMADS1 closer AGL24 using conserved ‘MADS’ domain. (B) Phylogenetic tree showing clustering of RcMADS1 closer AGL24 using conserved ‘K’domain. AGL24 and SVP from *Arabidopsis thaliana*; IbMADS3 and IbMADS4 from *Ipomoea batatas*; JOINTLESS from *Solanum lycopersicum*; MPF2 from *Physalis pubescens*; MPP3 from *Physalis peruviana*; PtMADS1 from *Populus tomentosa*; RcMADS1 from *Rafflesia cantleyi*; StMADS16 and StMADS11 from *Solanum tuberosum*.
(TIF)

Figure S5 **Phylogenetic tree of MADS-box proteins.** This consensus phylogenetic tree was generated via parsimony analysis using TNT version 1.0, with a data set based on the conserved MADS-box domain of approximately 60 amino acids. RcMADS1 is found to be nested within the StMADS11 clade (shown by arrow).
(TIF)

Figure S6 *RcMADS1* ectopic expression plant showing late-formed fertile flowers and siliques. (A) Whole plant, (B) close up of late-formed flowers and (C) close up of their siliques.
(TIF)

Figure S7 ***RcMADS1*** **ectopic expression plant with weak phenotype.** (A) Plant showing weak phenotype. (B) A silique with leaf-like sepals at the base.
(TIF)
