# Supplementary figures and images for: Identification and Characterization of RcMADS1, an AGL24 Ortholog from the Holoparasitic Plant Rafflesia cantleyi Solms-Laubach (Rafflesiaceae)
Source: PLoS One. 2013 Jun 28;8(6):e67243. doi: 10.1371/journal.pone.0067243 (PMC3695966; doi:10.1371/journal.pone.0067243)

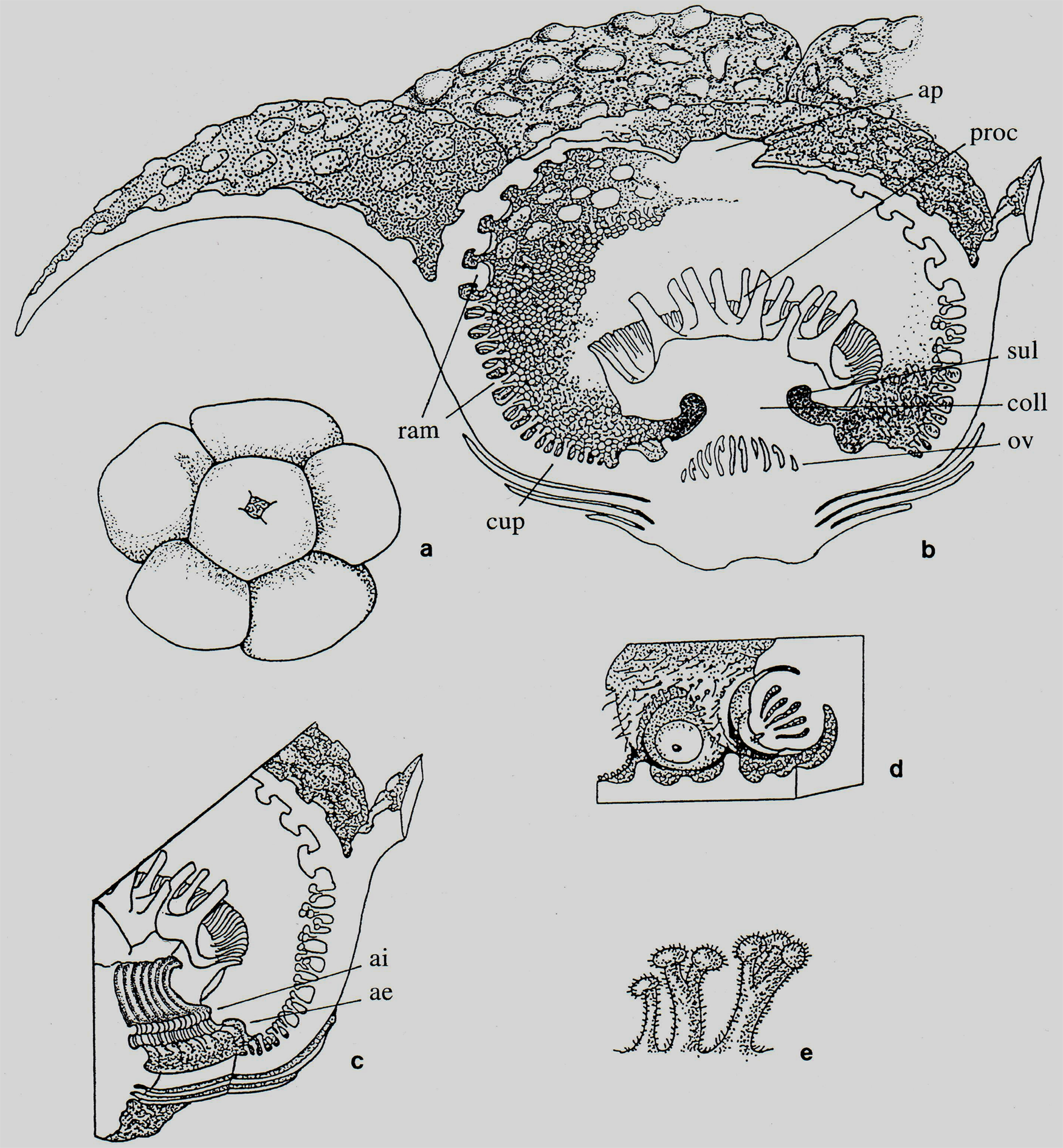

Supplement: Figure S1 — Different floral parts of Rafflesia micropylora Meijer. (a) Flower with the small aperture in the diaphragm. (b) Median longitudinal section view of female flower showing central column (ap: aperture in the diaphragm; coll: collum, neck of column; cup: cupula, perigone tube; ov: ovary; proc: processi on apex of disc; ram: ramenta on inside of cupula and diaphragm; sul: sulcus under disc. (c) Side view of the column showing outer and inner annulus (ae: annulus exterior; ai: annulus interior). (d) Section of male flowers, anther in longitudinal section and seen from lower side of the overhang of the ‘corona’ of the disc towards the sulcus. (e) Details of ramenta, often branched with swollen apices. This Figure is used with permission from Flora Malesiana Ser. I. Vol. 13 (1997). (TIF) [file pone.0067243.s001.tif]

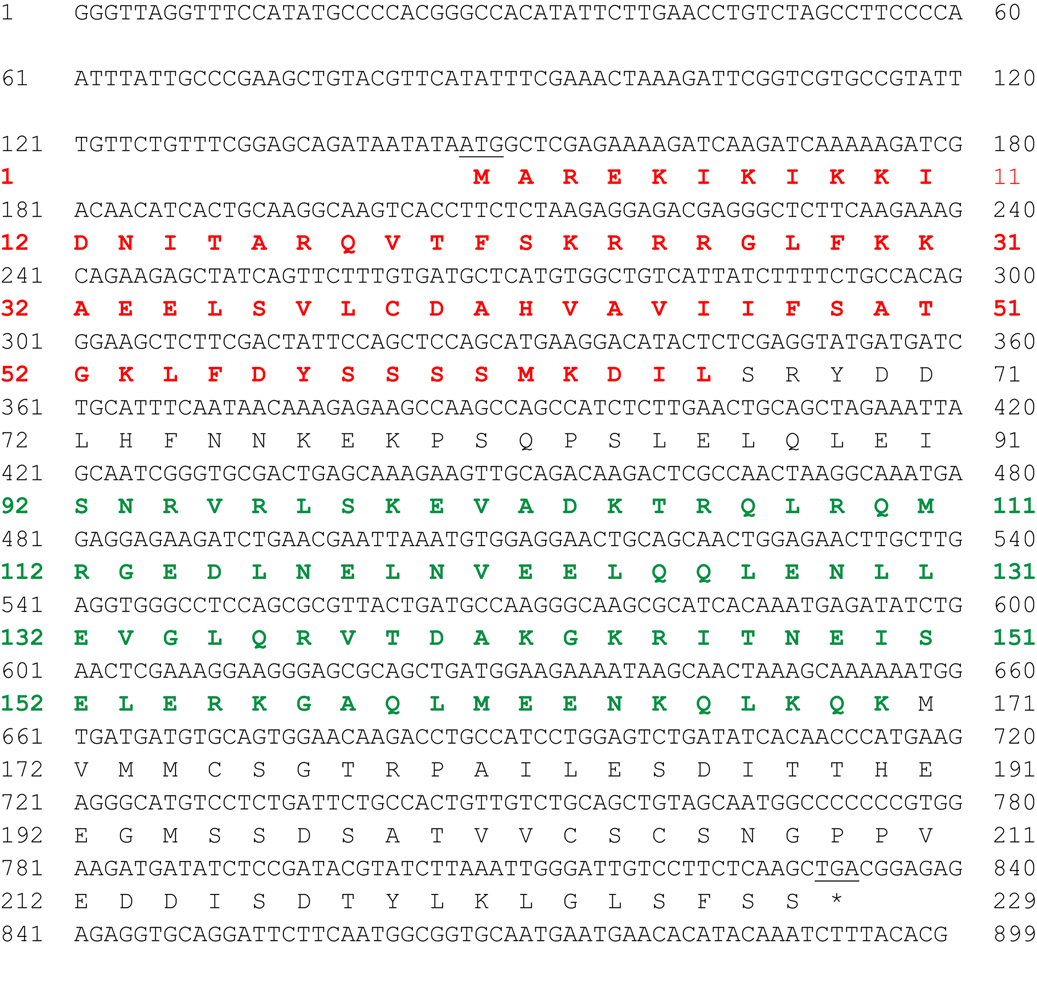

Supplement: Figure S2 — Sequence of RcMADS1 cDNA. The upper row is the nucleotide sequence, and the deduced amino acid sequence is in the lower row. The translation start (ATG) and termination (TGA) codons are underlined. The MADS-box and K domains are shown in red and green color, respectively. (TIF) [file pone.0067243.s002.tif]

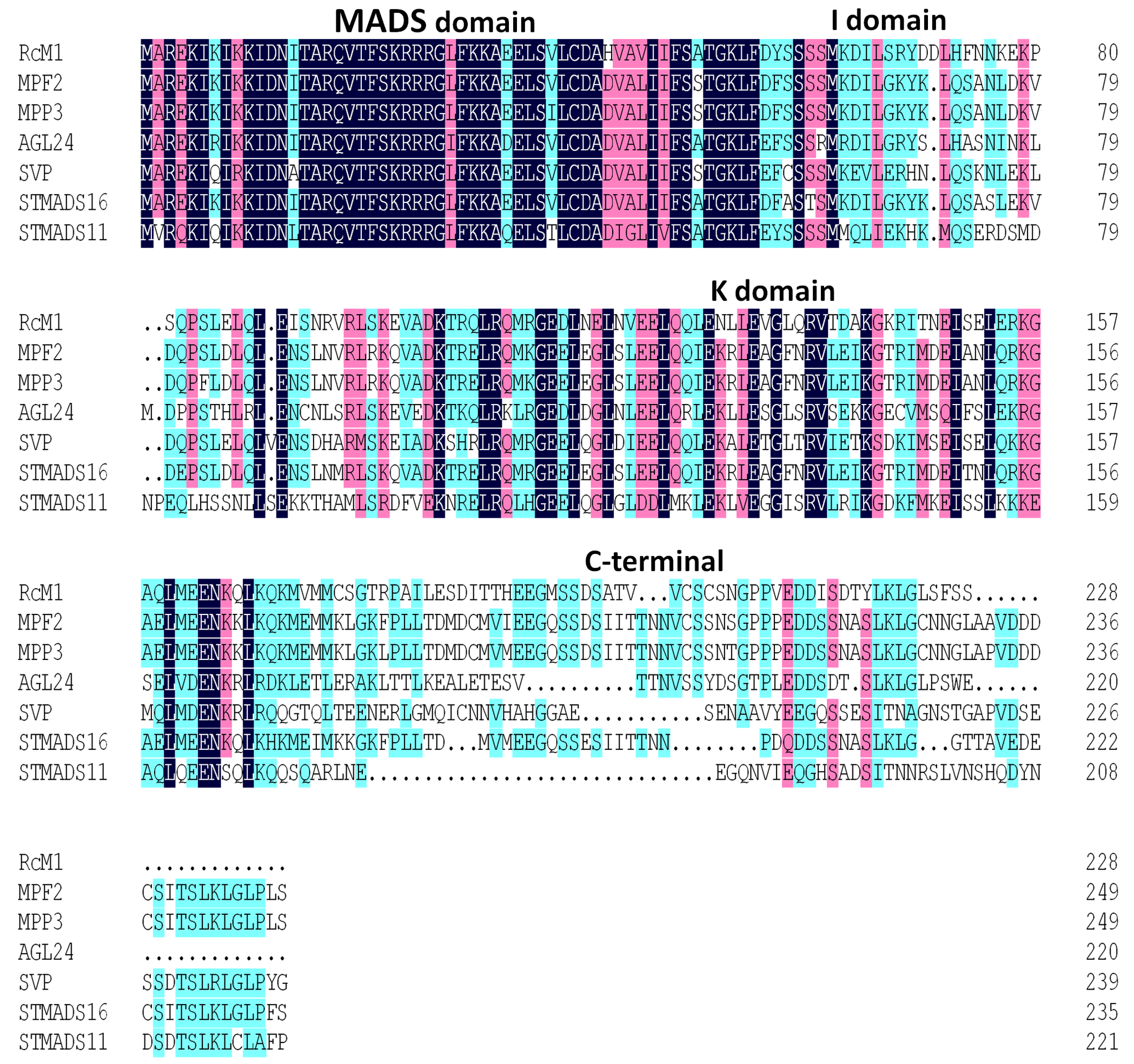

Supplement: Figure S3 — Alignment of the derived amino acid sequences of RcMADS1 and other members of the StMADS11 clade. The MADS, I, and K domains are all relatively conserved across the various proteins. Identical residues are coloured dark blue. Key to sequences included: RcM1 = RcMADS1 from Rafflesia cantleyi; MPF2 from Physalis pubescens; MPP3 from Physalis peruviana; AGL24 and SVP from Arabidopsis thaliana; and StMADS16 and StMADS11 from Solanum tuberosum. (TIF) [file pone.0067243.s003.tif]

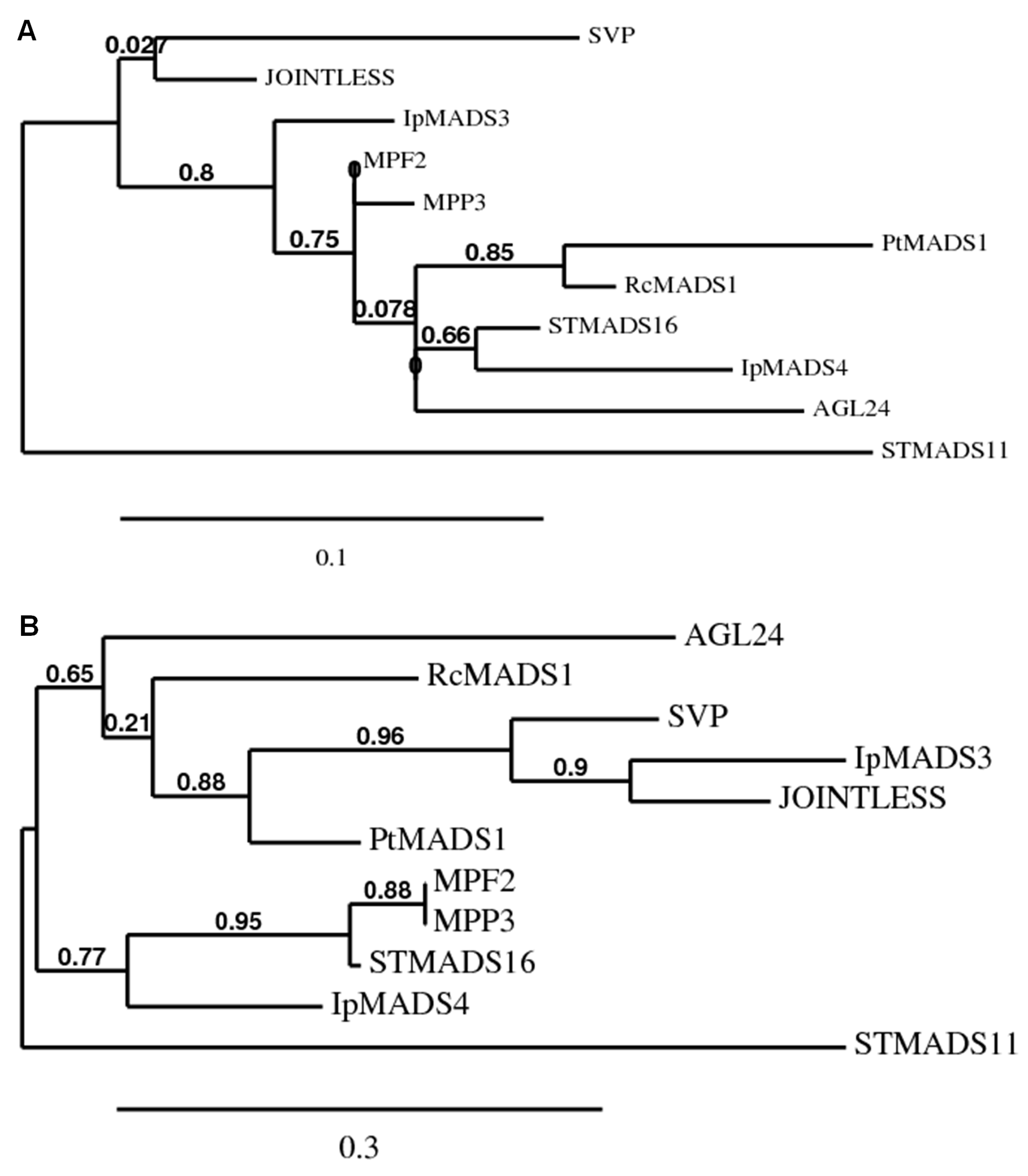

Supplement: Figure S4 — Phylogenetic tree of selected MADS-box genes from StMADS11 clade. (A) Phylogenetic tree showing clustering of RcMADS1 closer AGL24 using conserved ‘MADS’ domain. (B) Phylogenetic tree showing clustering of RcMADS1 closer AGL24 using conserved ‘K’domain. AGL24 and SVP from Arabidopsis thaliana; IbMADS3 and IbMADS4 from Ipomoea batatas; JOINTLESS from Solanum lycopersicum; MPF2 from Physalis pubescens; MPP3 from Physalis peruviana; PtMADS1 from Populus tomentosa; RcMADS1 from Rafflesia cantleyi; StMADS16 and StMADS11 from Solanum tuberosum. (TIF) [file pone.0067243.s004.tif]

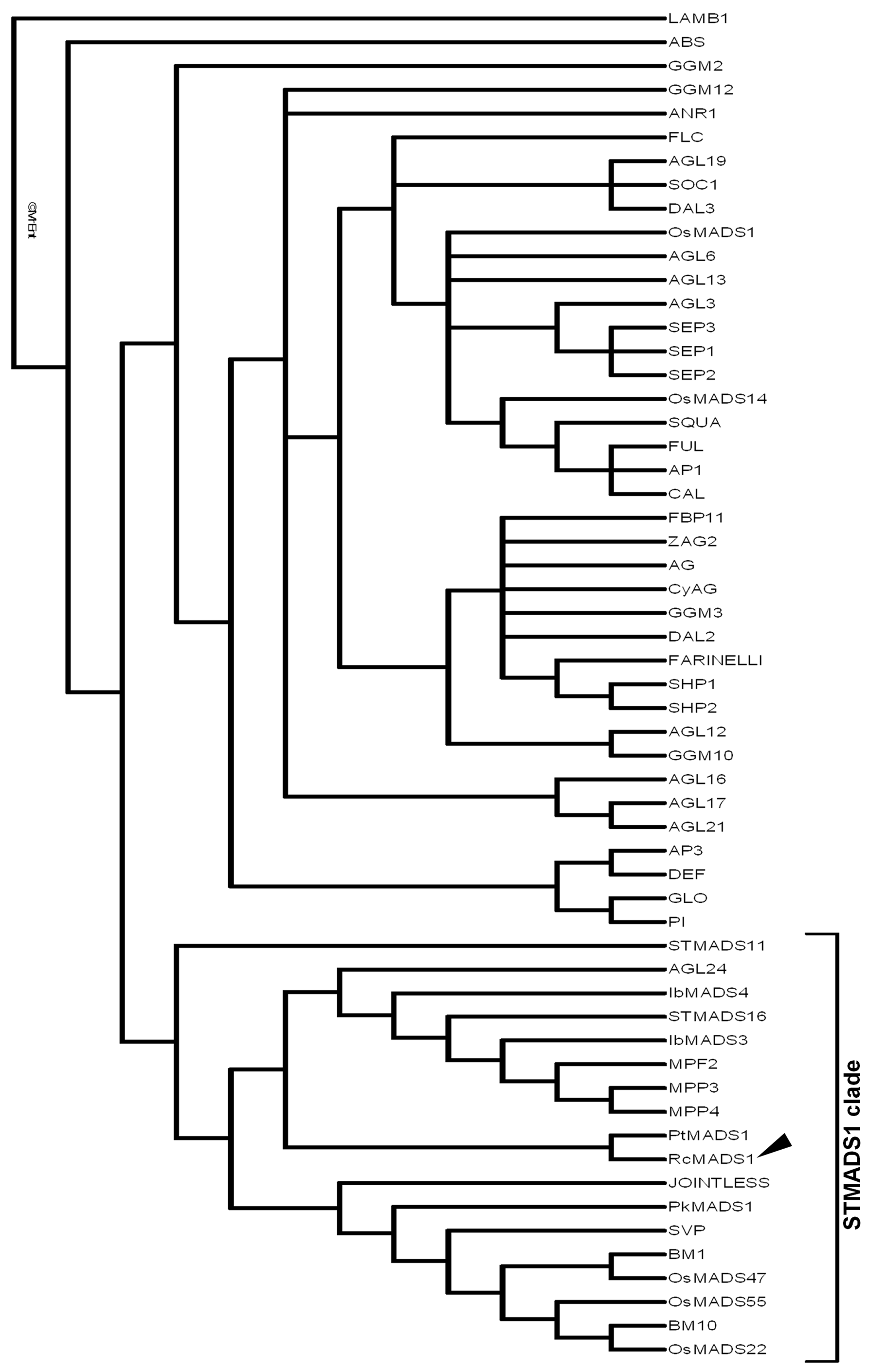

Supplement: Figure S5 — Phylogenetic tree of MADS-box proteins. This consensus phylogenetic tree was generated via parsimony analysis using TNT version 1.0, with a data set based on the conserved MADS-box domain of approximately 60 amino acids. RcMADS1 is found to be nested within the StMADS11 clade (shown by arrow). (TIF) [file pone.0067243.s005.tif]

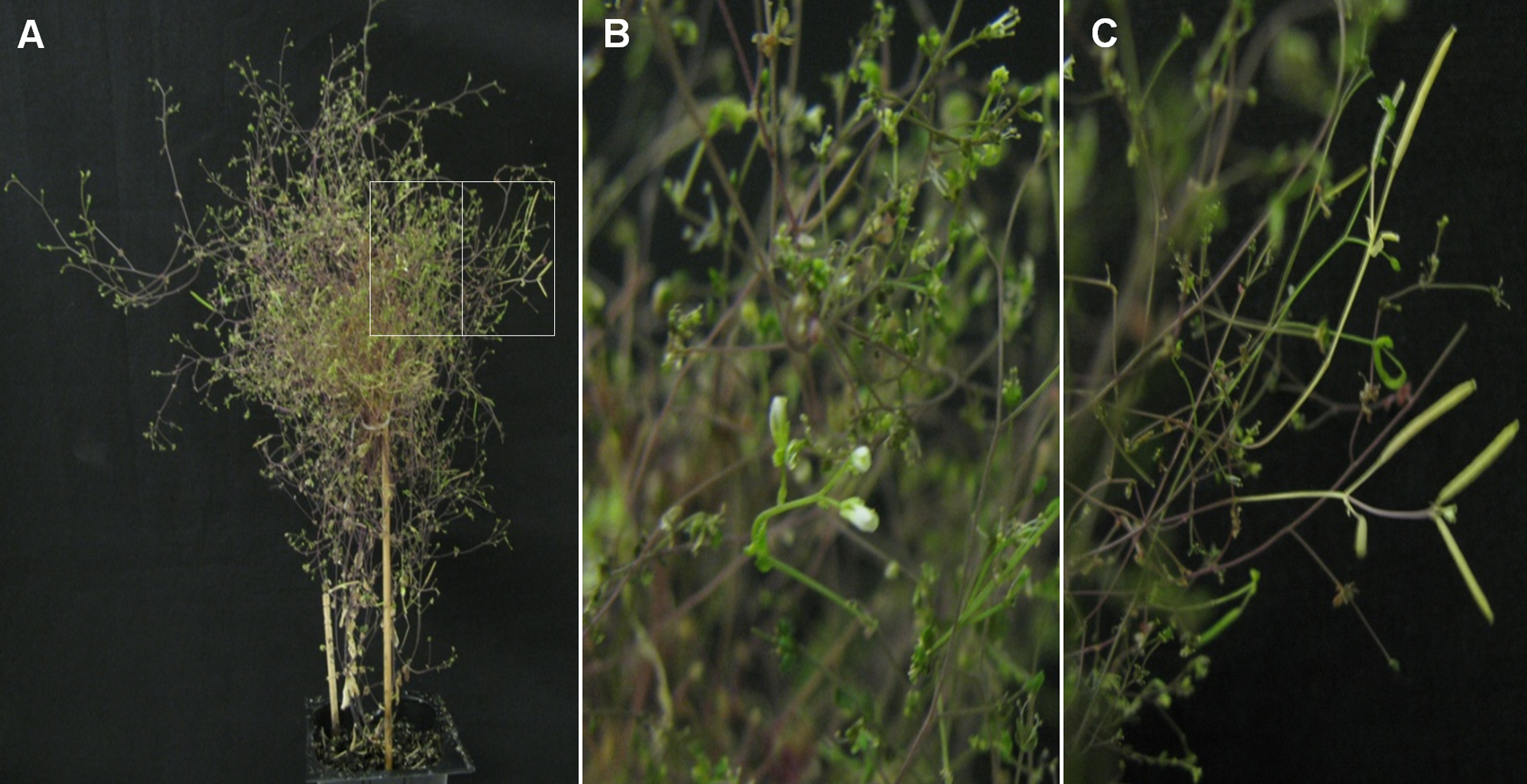

Supplement: Figure S6 — RcMADS1 ectopic expression plant showing late-formed fertile flowers and siliques. (A) Whole plant, (B) close up of late-formed flowers and (C) close up of their siliques. (TIF) [file pone.0067243.s006.tif]

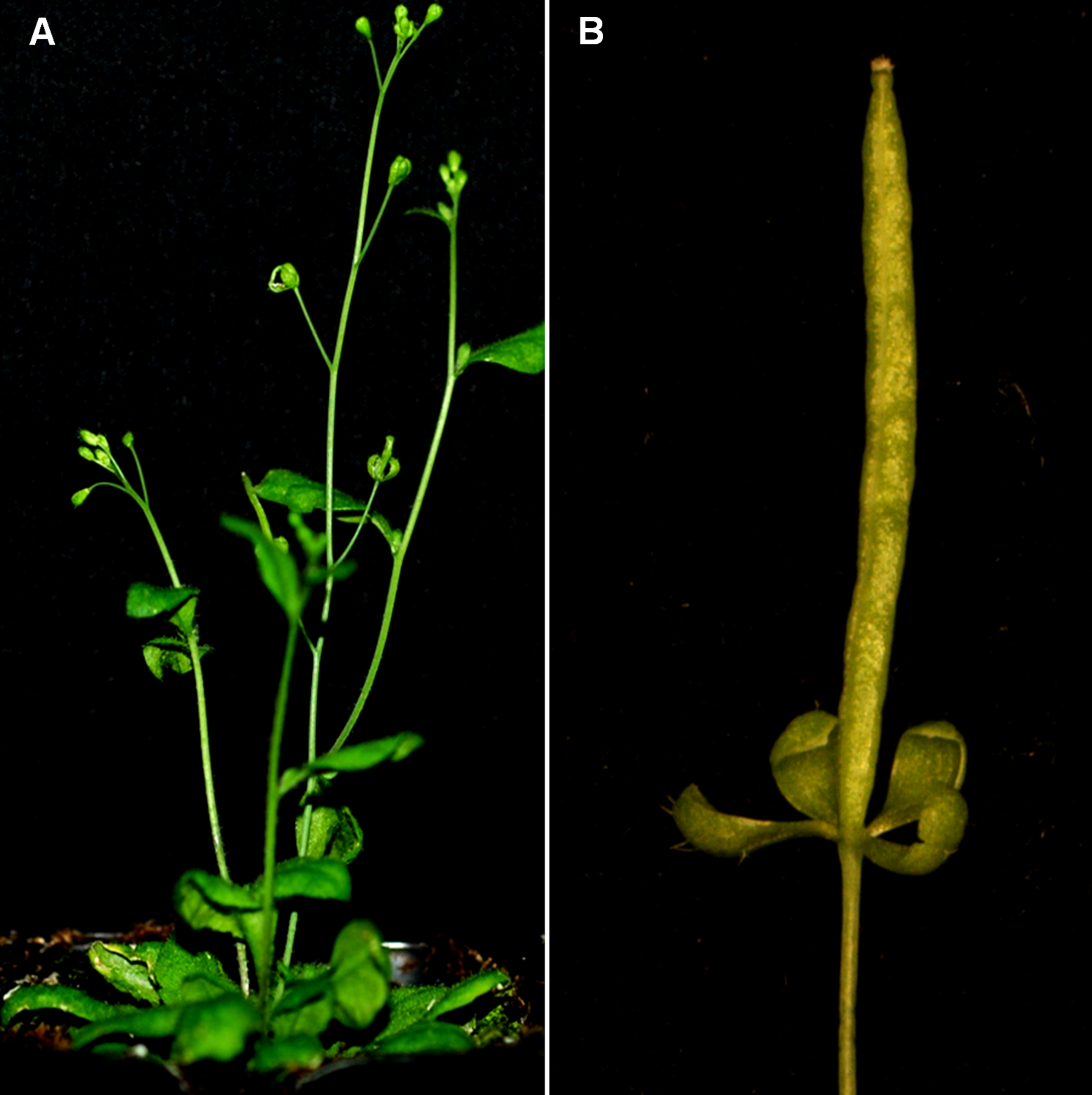

Supplement: Figure S7 — RcMADS1 ectopic expression plant with weak phenotype. (A) Plant showing weak phenotype. (B) A silique with leaf-like sepals at the base. (TIF) [file pone.0067243.s007.tif]
